# Bispecific antibody for lung cancer: mechanisms and clinical insights

**DOI:** 10.3389/fimmu.2025.1572802

**Published:** 2025-05-29

**Authors:** Wei Chen, Afang Zhou, Yunfeng Zhou

**Affiliations:** ^1^ Department of Thoracic Surgery, West China School of Public Health and West China Fourth Hospital, Sichuan University, Chengdu, Sichuan, China; ^2^ State Key Laboratory of Biotherapy, Sichuan University, Chengdu, Sichuan, China

**Keywords:** bispecific antibodies, lung cancer, novel therapies, immunotherapy, targeted therapy

## Abstract

Lung cancer is a refractory malignancy. Although various therapeutic options, including targeted therapies, immune checkpoint inhibitors, and systemic chemotherapy, have significantly improved the prognosis of lung cancer patients, five-year survival rates are still low. Bispecific antibodies have attracted much attention because of their ability to bind different antigens or epitopes on the same antigen at once and because of their multiple novel functional mechanisms. Recently, three bispecific antibodies have been successively approved for lung cancer treatment, demonstrating the potential of bispecific drugs in lung cancer therapy. Various bispecific antibodies are currently under clinical trials to evaluate their safety and efficacy in lung cancer. In this review, we provide an overview of these antibodies’ structure and mechanism of action, summarize their clinical progress in lung cancer treatment, and discuss and analyze the challenges and future directions of bsAbs application in lung cancer.

## 1 Introduction

Lung cancer is one of the world’s most common cancers and the leading cause of cancer-related deaths, with an estimated 2.2 million new cases and 1.79 million deaths annually (1). In most parts of the world, the 5-year survival rate for lung cancer patients is only 10-20% (2). Non-small cell lung cancer (NSCLC) is one of the most common types of lung cancer, accounting for about 85% of lung cancers (1). The treatment landscape for NSCLC has changed dramatically over the past decade by introducing several new targeted and immunotherapeutic agents. Patients treated with protein kinase inhibitors [especially tyrosine kinase inhibitors (TKIs)] and monoclonal antibodies [e.g., immune checkpoint inhibitors (ICIs)] may have a relatively good prognosis. However, although the above treatment strategies significantly prolong the overall survival of patients, a common problem is drug resistance (3, 4). Small cell lung cancer (SCLC) is another type of lung cancer that accounts for 10-15% of all lung cancers (5–7). It is even a tumor type with an inferior prognosis and limited therapeutic options, with a median survival of 2 years for most patients with early-stage disease and 1 year for patients with metastatic disease (8). Therefore, there is still a significant unmet medical need in the field of lung cancer.

Bispecific antibodies (bsAbs) have been described as “next-generation antibodies” that overcome the limitation of natural monoclonal antibodies to bind only a single epitope (9). Amivantamab is the first bispecific antibody approved for the treatment of lung cancer. The drug was initially approved for treating adult patients with locally advanced or metastatic NSCLC harboring epidermal growth factor receptor (*EGFR*) Exon 20 insertion mutations whose disease has progressed on or after platinum-based chemotherapy (10). Several bsAbs with potential for lung cancer therapy are currently undergoing clinical trials, and many have produced exciting results. This review provides an overview of the bsAbs that have shown promise in treating lung cancer.

## 2 Bispecific antibody formats and mechanisms

**Figure 1:**
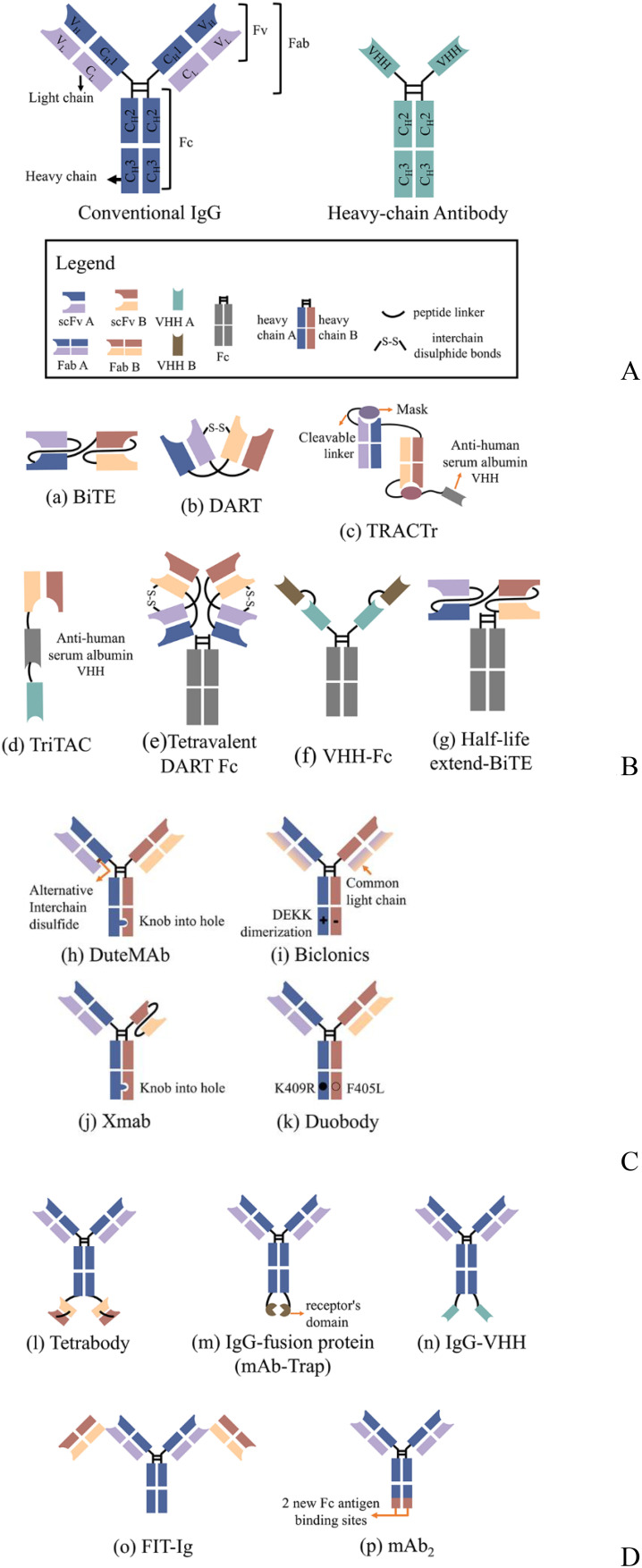
Schematic overview of the antibody structure and representations of several classical bsAbs formats; **(A)** Conventional IgG consists of 2 heavy chains and two light chains, while heavy-chain antibodies are only comprised of heavy chains; **(B)** No IgG-like bsAbs; those bsAb consist of antibody-based fragments, such as scFv, VHH, Fab, Fc; **(C)** Asymmetric IgG-based bsAbs; the molecules may contain mutations, knob-into-hole or DEKK for example, that affect chain pairing and other manufacturability parameters. **(D)** Symmetric IgG-based bsAbs; Symmetric bispecific antibodies are generated by a fusion of an additional binding site to the heavy/light chains or by making differential but overlapping use of the light and heavy complementarity determining regions as primary contacts for each antigen. scFv, single-chain variable fragment; Fab, antigen-binding fragments; VHH, variable heavy domain of heavy chain; Fc, fragment crystallizable.

One of the significant challenges of dual antibodies, which took about half a century to move from concept to the clinic, is that only 12.5% of the target molecules can be obtained by conventional production means, with the rest being mostly nonfunctional or monospecific molecules (11, 12). To address this challenge, researchers have developed various strategies based on natural antibodies such as IgG and heavy-chain antibodies (**Figure 1A**). These strategies aim to increase the proportion of the target molecule and facilitate its separation and purification, while enabling the modular combination of distinct antibody functional domains as required. Today, more than 100 types of bsAbs are known (9) and can be briefly classified into three categories: no IgG-like bsAbs (**Figure 1B**), asymmetric IgG-based bsAbs (**Figure 1C**), and symmetric IgG-based bsAbs (**Figure 1D**). No IgG-like bsAbs consist of combinations of partial structures of antibodies. Among them, bsAbs designed based on single-chain variable fragment (scFv) (e.g., BiTE, DART, etc.) are the simplest and the least difficult to generate. Moreover, due to their low molecular weight, they have better tissue permeability. However, the absence of fragment crystallizable (Fc) structure results in a short plasma half-life of these molecules and a lack of Fc-mediated effector function [e.g., antibody-dependent cell cytotoxicity (ADCC) or antibody-dependent cell phagocytosis (ADCP)]. To extend the half-life of such bsAbs, a common strategy is to fuse Fc fragments (including HEL-BiTE, Tetravalent DART Fc, VHH-Fc, etc.) or conjugate fragments of anti-human serum albumin (HSA) antibodies (e.g., TRACTr, TriTAC) with the bsAbs protein. IgG-based bsAbs retain Fc and have a longer half-life, and Fc function can be adjusted to enhance the therapeutic effect of the molecule according to specific needs. However, there will be more factors to consider in the molecular design of the protein. For example, asymmetric IgG-based antibodies are closer in form to natural IgG. However, as mentioned above, the target molecule content is only 12.5% when produced by conventional means, and it is not easy to purify the target molecule from the system to obtain a high-purity target molecule. Therefore, a series of molecular modifications are needed to promote the correct pairing of light and heavy chains to increase the target molecule content, and these strategies include knob-into-hole, DEKK mutation, common light chain, and the addition of alternative interchain disulfide. Symmetric IgG-based bsAbs are made by fusing another antigen-binding fragment to the conventional antibody (e.g., Tetrabody, IgG-VHH, FIT-Ig, etc.) or by mutating Fc to form a new antigen-binding site (e.g., mAb2) and thus do not need to consider the correct pairing of light and heavy chains. However, such modifications can change physicochemical properties such as antibody stability and solubility (13, 14). In addition, antigen-antibody binding is affected by the position of the fused fragment (15).

## 3 The mechanisms of action of bispecific antibodies in lung cancer

**Figure 2:**
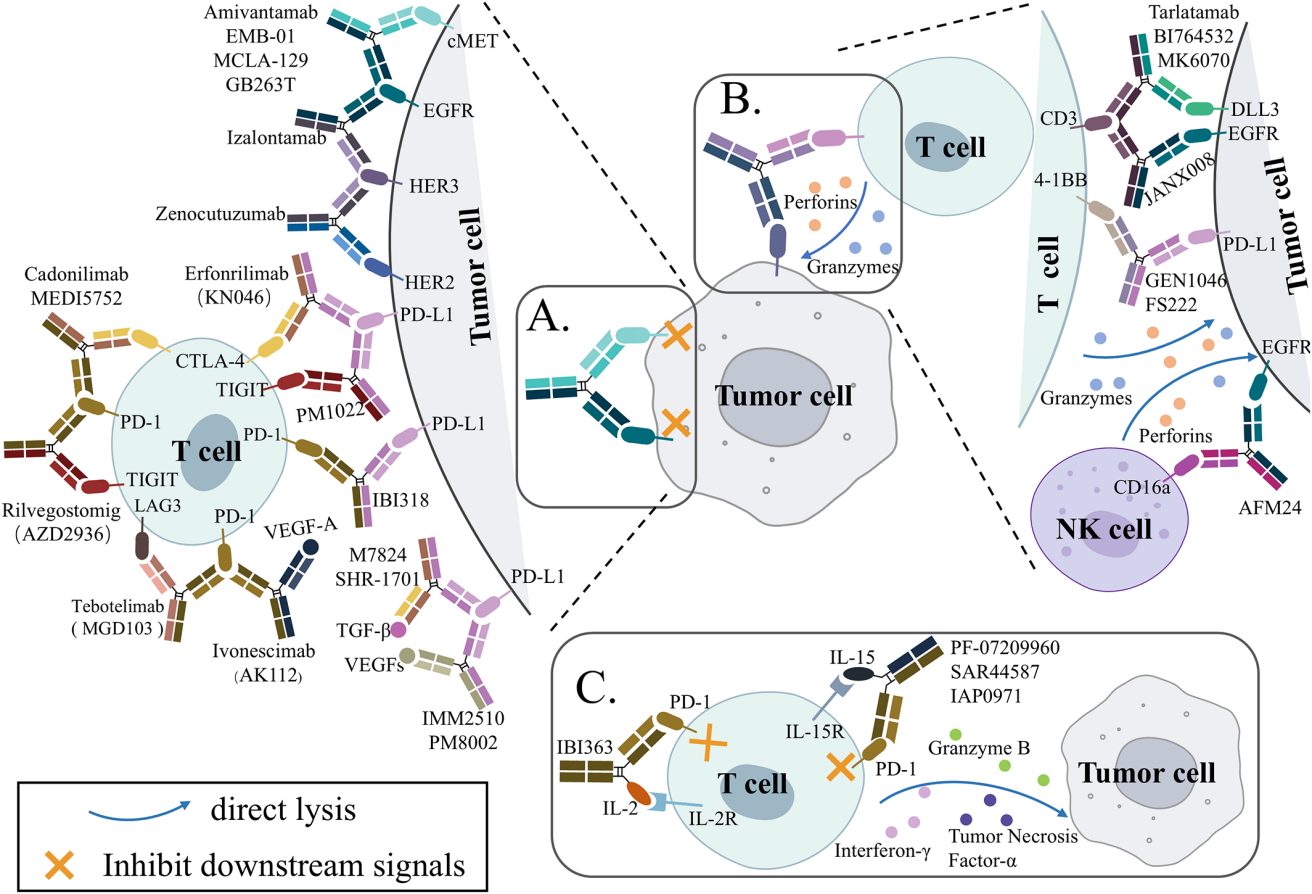
Simplified schematic overview of the proposed mechanisms of action for bispecific antibodies (bsAbs) in clinicals for lung cancer treatment. **(A)** Blocking signaling. Two targets are being disrupted by the bsAb. **(B)** Engagement of immune cells to the tumor cell. Immune cells can be engaged to tumor cells by bsAbs. **(C)** Immunocytokine. Increase cytokine accumulation within the tumor and block immune checkpoint. cMET, mesenchymal-epithelial transition; EGFR, epidermal growth factor receptor; HER2, human-epidermal growth factor receptor 2; HER3, human-epidermal growth factor receptor 3; PD-1, programmed cell death protein 1; PD-L1, programmed death-ligand 1; CTLA-4, cytotoxic T-lymphocyte-associated protein 4; TIGIT, T cell immunoglobulin and ITIM domain; LAG-3, lymphocyte activation gene 3; VEGF, vascular endothelial growth factor; TGF-β, transforming growth factor β; DLL3, Delta-like ligand 3; IL-2, interleukin-2.

Unlike the simple mixing of antibodies, bsAbs have become a primary focus of drug developers because they have new mechanisms of action (MOA) different from those of the parent antibody combination. Currently, bsAbs used in lung cancer therapy include three main mechanisms: dual inhibition (**Figure 2A**), engaging immune cells and tumor cells (**Figure 2B**), and immune cytokines (**Figure 2C**).

By targeting two antigens at the same time, dual-inhibition bsAbs inhibited the pathways of two signals that are related to each other, exerting the effect of 1 + 1>2. More than half of the bsAbs currently applied in lung cancer treatment mainly exert anti-tumor effects by this mechanism of action. They can be further categorized into three types according to the difference in the signals they block: (i) simultaneous targeting of two surface receptors with specific signaling and functional overlap associated with tumorigenesis and progression, mainly ErbB family proteins; (ii) dual immune checkpoint molecule blockade; (iii) simultaneous inhibition of immune checkpoints and tumor microenvironmental pro-tumor growth factors.

Immune cell engagers (ICEs) redirect cytotoxic immune cells to disease-associated target cells that play a key role in the disease process to achieve direct killing of these cells by immune cells. Among them, T cell engagers (TCEs) are the typical application of bsAb, and about half of the bsAbs currently evaluated in clinical trials are TCEs (16). Recently, researchers have also been experimenting with Natural Killer cell engagers (NKCEs) based on the recruitment of cytotoxic NK cells (17, 18).

Immunocytokines are a type of antibody-cytokine fusion protein. Mechanistically, immunocytokines fuse a therapeutic cytokine to one end of an antibody. Through the specific targeting function of the antibody, the cytokine is specifically delivered near tumor cells, allowing it to bind to specific cytokine receptors on the surface of surrounding immune cells, thus significantly reducing non-specific toxicity (19, 20). Structurally, immunocytokines have a symmetric or asymmetric structure based on IgG (21). Therefore, in some literature and this article, immunocytokines are classified as a type of bispecific antibody with a special mechanism (22, 23).

The following is a further discussion and analysis of bispecific antibodies in lung cancer according to the different mechanisms of action described previously.

## 4 Dual inhibition bispecific antibodies

### 4.1 Dual receptors inhibition bispecific antibodies

The ErbB family of transmembrane receptor tyrosine kinases consist of four members: EGFR/ErbB1/HER1, ErbB2/Neu/HER2, ErbB3/HER3, ErbB4/HER4. ErbB receptors are activated upon homo-or heterodimerization, which activates many downstream signaling pathways, primarily the mitogen-activated protein kinase (MAPK), phosphatidylinositol 3-kinase (PI3K)/protein kinase B (PKB/AKT), and Janus kinase (JAK) and signal transducer/activator of transcription (STAT) signaling pathways (24). Moreover, all these pathways regulate cell metabolism, growth, and survival. Overexpression and overactivation of ErbB receptors are associated with poor prognosis, drug resistance, tumor metastasis, and lower survival in a variety of cancers, including lung cancer. There are two clinically important ErbB inhibitors: humanized antibodies targeting the extracellular structural domains of EGFR or HER2 and small-molecule TKIs that compete with adenosine triphosphate in the structural domain of the receptor tyrosine kinase. Eventually, however, a significant proportion of tumor cells develop resistance through the activation of another ErbB receptor signal or the activation of bypass pathways (25, 26). BsAbs inhibit tumor growth more effectively by simultaneously targeting two ErbB members or related bypass pathways, thereby blocking overlapping downstream signals.

#### 4.1.1 EGFR × cMET

*EGFR* mutations exist in approximately 50% of Asian NSCLC patients and 11-16% of European NSCLC patients (27–29). Currently, the US Food and Drug Administration (FDA) has approved six EGFR TKIs, as well as a fully humanized monoclonal antibody targeting EGFR, as the standard of care for the first-line treatment of NSCLC patients with *EGFR* mutations (30). However, the selection pressure exerted by the above drugs inevitably leads to treatment resistance. Among cases of acquired resistance to EGFR TKIs, 5-10% are mesenchymal-epithelial transition factor (*MET*) amplified, which activates the EGFR-independent PI3K-AKT signaling pathway by driving ErbB3 dimerization and signal transduction (31, 32). Drug-resistant tumors are also able to activate the cMET pathway through increased cMET expression and/or increased cMET ligand expression, which provides an alternative mechanism for tumor cells to bypass the TKI blockade of EGFR and promote cancer cell survival (33–36). Due to the signaling crossover between EGFR and cMET, combined inhibition of both receptors may limit the activation of the compensatory pathway and improve overall efficacy.

Amivantamab (JNJ-61186372; Rybrevant™) is a fully humanized bsAb targeting EGFR and cMET and is the first approved therapeutic agent for NSCLC patients with *EGFR* exon 20 insertion (*EGFR* ex20ins) after failure of platinum-containing chemotherapy. In addition to its ability to block both EGFR- and cMET-mediated downstream signaling, the antibody exerts its anti-tumor effects through various Fc-mediated mechanisms, such as ADCC and ACDP (37, 38). The CHRYSALIS (NCT02609776) analyses the efficacy and safety of amivantamab in post-platinum NSCLC patients with *EGFR* Exon20ins. In the efficacy population, the reported overall response rate (ORR) was 40%, the median duration of response (mDOR) was 11.1 months, and the median overall survival (mOS) was 22.8 months, respectively (10, 39, 40). Besides, the drug has a favorable safety profile, with the most common side effects including rash (89%) and infusion-related events (67%). Based on the above data, the FDA approved the new drug application of amivantamab in 2021 (41). In a subsequent clinical trial called PAPILLON (NCT04538664), researchers analyzed the anti-tumor activity of amivantamab in combination with carboplatin-pemetrexed. Among treatment-naïve NSCLC patients with *EGFR* ex20ins, the progression-free survival (PFS) in the amivantamab plus chemotherapy group was significantly longer than that of the chemotherapy group (median, 11.4 months and 6.7 months, respectively) (42). Based on this clinical result, the FDA approved amivantamab plus chemotherapy as a first-line therapy for advanced NSCLC with *EGFR* ex20ins mutation (43).

The efficacy of amivantamab is not limited to *EGFR* ex20ins mutation. However, it has shown positive clinical benefits for the larger population of patients with other *EGFR* and *MET* mutations. For patients with locally advanced or metastatic NSCLC with *EGFR* mutations (Ex19del or L858R) (NCT06120140), compared to those receiving osimertinib, patients treated with amivantamab plus lazertinib had longer mPFS (23.7 months vs. 16.6 months, HR=0.7) and longer mDoR (25.8 months vs. 16.8 months) (44, 45). For primary *MET*ex14 patients with advanced NSCLC treated with amivantamab (NCT02609776), an ORR of 33% (56% in the treatment-naïve population) with a mDOR of 11.2 months was observed (46). In addition, a subcutaneous formulation of amivantamab, based on human hyaluronidase, was developed to improve patient tolerability and reduce administration time (47). It was shown (NCT05388669) that when administered subcutaneously, the drug was not only pharmacokinetically non-inferior to intravenous administration, but fewer patients in the subcutaneous group experienced infusion-related reactions (13% vs. 66%) and venous thromboembolism (9% vs. 14%). Concurrently subcutaneously administered patients had prolonged mPFS (6.1 months vs. 4.3 months, HR=0.84), prolonged mDOR (11.2 months vs. 8.3 months), and significantly prolonged OS (HR=0.62).

EMB-01 is an EGFR- and cMET-targeted bispecific antibody based on the FIT-Ig technology platform, which fuses the Fab of an anti-cMET antibody to the variable region of an anti-EGFR antibody to form a tetravalent bispecific antibody. EMB-01 induces endocytosis of EGFR and cMET receptors on the cell surface and their degradation. Preliminary clinical (NCT03797391) data suggest an ORR of 5.3% and a DCR of 42.1% in 38 evaluable patients with advanced NSCLC (48).

MCLA-129 is a 1 + 1 form of asymmetric Ig-G-like bispecific antibody designed based on the biclonics common light chain platform (49). It targets both EGFR and cMET and has a mechanism of action similar to amivantamab. Preliminary clinical data (NCT04930432) on fortnightly intravenous administration of 1,500 mg MCLA-129 in different NSCLC patients were recently published: for patients with *MET*ex14 mutation, the ORR was 43.5%, and the DCR was 95.7%; for *EGFR*20ins-mutated patients, the ORR was 28.6% and DCR of 84.1%; for patients with sensitized *EGFR*-mutated, ORR was 21.8% and DCR was 69.1% (50). In addition, this bispecific antibody in combination with osimertinib was observed in treatment-naïve patients with advanced *EGFR*mut NSCLC (NCT04868877) with an ORR of 75.0% and a DCR of 93.8%; in patients who progressed on osimertinib, the ORR was 35.3%, and the DCR was 73.5% (51).

#### 4.1.2 HER2 × HER3

As a member of the ERBB receptor family, HER2 alterations are involved in the oncogenic process in a variety of solid tumors, mainly including *HER2* mutation, *HER2* amplification, and HER2 overexpression, with corresponding incidence rates of 1%-6.7%, 2%-22%, and 7.7%-23%, respectively, in NSCLC and all of them are associated with poor prognosis (52–55). HER3 is overexpressed in 83% of primary NSCLC tumors and is associated with advanced disease, shorter time to metastasis, and lower survival (56). HER2 is activated by dimerization or with other ERBB family members and further activates downstream signaling pathways. Of all possible EGFR family dimers, the HER2:HER3 heterodimer has the highest translational capacity (57–59). When HER3 binds to its ligands (neuregulin1-4, NRG1-4), its conformation is altered, exposing its dimerization sites with other proteins of the EGFR family, predominantly EGFR and HER2, which induces phosphorylation events downstream of the protein (60). Notably, NRG1 can form fusion proteins with various membrane proteins, which provide a transmembrane structural domain to anchor NRG1 to the membrane, thus enabling NRG1 to bind to HER3 in its own or neighboring cells (61). NRG1 fusions have been detected in a wide range of tumors, with the highest number of cases reported in NSCLC (62). Early reports observed relatively poor NRG1 fusion-positive (NRG1^+^) lung therapy outcomes. The ORR of those patients treated with platinum-doublet and taxane-based (post-platinum-doublet) chemotherapy was only 13% and 14%, with mPFS of 5.8 and 4.0 months, respectively (63).

Zenocutuzumab (MCLA-128) is an IgG-like asymmetric bsAb targeting HRE2 and HER3. The antibody preferentially binds to more abundant HER2 proteins on the cell surface via the higher affinity HER2-targeting arm, providing a high concentration of local antibody while at the same time positioning the HER3-targeting arm to block NRG1 or NRG1 fusion proteins binding to HER3 (64). Thereby, the formation of HER2:HER3 heterodimers is potently inhibited, preventing subsequent phosphorylation of the HER3 cytoplasmic structural domain and downstream oncogenic signaling. In addition, glycoengineering modifications enhanced the antibody’s ADCC activity (64). The FDA recently granted accelerated marketing approval for zenocutuzumab based on clinical results from a study called eNRGy (NCT02912949) (65). In 64 evaluable patients with NRG1^+^ advanced NSCLC, the confirmed ORR was 34% (22/64; [95% CI] 23-47), and the mDOR was 12.9 months, with responses ongoing in 11/22 (50%) patients (66). The drug’s safety profile was favorable, with <4% of patients experiencing grade ≥3 adverse events (66).

#### 4.1.3 EGFR × HER3

In addition to HER3 forming dimers with HER2 to deliver proliferation and survival signals to the cells, another important dimerization partner is EGFR. It has been found that regardless of the type of EGFR-TKIs resistance mechanisms, such as *EGFR* T790M mutation, *MET* amplification, and *HER2* amplification, *HER3* amplification is observed in *EGFR*-mutated NSCLC tumors that progress after EGFR-TKI treatment (67). HER3 can also be activated independently of ligand binding through dysregulation of other tyrosine kinase receptors. For example, in NSCLC carrying activating *EGFR* mutations, EGFR can transactivate HER3 via heterodimers (68). In the presence of EGFR-TKIs, *MET* amplification activates HER3, thereby initiating a downstream PI3K/AKT survival mechanism (31).

Izalontamab (SI-B001) is a tetravalent symmetric bsAb targeting EGFR and HER3. The antibody is cetuximab-based and contains an anti-HER3 scFv at the end of the constant region of cetuximab (69). Since the antibody has a significantly lower affinity for the HER3 arm than the EGFR arm, the antibody can only bind to HER3 after definitive binding to the EGFR (69). As a result, it effectively inhibits tumor cells that express both EGFR and HER3, minimizing the effect on functioning HER3 in normal tissues. In a phase II clinical trial (NCT04603287), researchers enrolled 55 patients with locally advanced or metastatic *EGFR/*anaplastic lymphoma kinase (*ALK*) wild-type NSCLC who had failed first-line anti-PD-1/L1 therapy with or without platinum-based chemotherapy (PBC) to receive SI-B001 combined with docetaxel (70). Of the 48 evaluable patients, the ORR and the DCR were 31.3% and 77.1%, respectively (63). Among the patients in Cohort B who failed first-line anti-PD-1/L1 combined with PBC, 22 of the evaluable patients in this cohort were on a regimen of 16 + 9 mg/kg/week. These patients’ reported ORR and DCR were 45.5% and 68.2%. Among all, the most common≥grade 3 treatment-related adverse events (TRAEs) were myelosuppression (17%), decreased neutrophil count (15%), and decreased white blood cell count (12%).

### 4.2 Dual immune checkpoints inhibition bispecific antibodies

The immune system is an elaborate and complex network of multiple signals that activate immune cells to accurately recognize and eliminate pathogenic microorganisms and mutated cells in the body. At the same time, to avoid damage to normal tissues and organs by excessive immune response, the immune system has evolved a series of checkpoints to modulate the duration and amplitude of the immune response. However, tumor cells will hold these checkpoints hostage to escape immune surveillance (71). Reactivating or enhancing the immune system’s innate or adaptive immune response to strengthen the attack on cancer cells is an important strategy for cancer treatment.

#### 4.2.1 PD-1/L1 × CTLA-4

Programmed cell death protein 1 (PD-1), programmed death-ligand 1(PD-L1), and cytotoxic T-lymphocyte-associated protein 4 (CTLA-4) are the relatively well-studied and most maturely applied immune checkpoints. Currently, immune checkpoint inhibitors are approved for treating various solid and hematological malignancies (72). Despite the success of anti-CTLA-4 and anti-PD-1/L1 therapies, only 10-25% of patients benefit from such treatments (73). Because of the similarities and differences between the CTLA-4 and PD-1 pathways and some complementarity between them (74), combination therapy with anti-PD-1/L1 and anti-CTLA-4 clinically improves overall survival. The combination of these two classes of antibodies has been approved for the treatment of various tumors, including non-small cell lung cancer. However, at the same time, the toxicities of the combination are more intense than those of the single agent (75–79). On the other hand, PD-1 and CTLA-4 are co-expressed in a high percentage of tumor-infiltrating lymphocytes (80). Therefore, it is possible to maximize clinical benefit and minimize additional toxicity by designing bispecific antibodies that can specifically target such tumor-infiltrating lymphocytes and avoid activation of immune cells in other normal tissues by the bsAbs.

Cadonilimab (AK104; 开坦尼^®^) is a humanized tetravalent symmetric bispecific antibody incorporating an anti-CTLA-4 scFv fragment at the C-terminus of each of the two heavy chains of the anti-PD-1 antibody (81). Also, to avoid T-cell depletion and adverse immune responses caused by Fc-mediated immune cell activation, the Fc segment was mutated to completely remove ADCC, ADCP, and CDC effects (81). Cadonilimab has a higher affinity at high PD-1 concentrations and a relatively low binding capacity at lower PD-1 concentrations (82). As a result, the antibody has higher activity in high PD-1-expressing tumor microenvironments and weaker activity in normal tissues. It received conditional approval for marketing in China in June 2022 for the treatment of patients with recurrent or metastatic cervical cancer who have failed prior treatment with platinum-containing chemotherapy (83). Several clinical trials of cadonilimab alone or combined with other drugs for treating NSCLC and SCLC are underway (83). A clinical trial called AK104-208 (NCT04646330) evaluated the efficacy of cadonilimab in combination with anilotinib, an anti-angiogenic drug, in treatment-naïve patients with NSCLC. In 69 evaluable patients, the overall ORR was 53.6% (95% CI, 41.2-65.7), the DCR was 92.8% (95% CI, 83.9-97.6), and grade 3^+^ TRAEs occurred in 49.3% of patients (84). Another study called AK104-IIT-018 (NCT05816499) included patients with histologically or cytologically confirmed stage IIIB/IIIC or IV NSCLC without sensitizing *EGFR/ALK/ROS1* mutations, who must have progressed during or after a PD-1/L1 inhibitor and platinum-based chemotherapy and were treated with three-drug combination therapy (cadonilimab plus anilotinib plus docetaxel) (85). Among 33 evaluable patients, the overall ORR was 30.3% (95% CI:15.6-48.7%), the DCR was up to 94.0% (95% CI:79.8-99.3%), the mPFS was 6.5 months, and the proportion of ≥ grade 3 TRAEs was only 17.4% (85). Patients with advanced driver-negative NSCLC have limited therapeutic options after progression on first-line immune-combination chemotherapy (86). The standard of care recommended by the NCCN guidelines is chemotherapy monotherapy, such as docetaxel, gemcitabine, and albumin-conjugated paclitaxel. However, the efficacy was minimal, with ORR of 14%-17% and mPFS of 4.0-5.4 months (87–89). Therefore, combining cadonilimab with anlotinib and docetaxel offers a potentially attractive treatment option for this group of patients. The LungCadX study (NCT06424821)evaluated the efficacy and safety of cadonilimab in combination with chemotherapy as first-line treatment for patients with driver-negative, PD-L1-negative advanced NSCLC (90). Among 30 evaluable patients, the overall ORR was 66.7%, with an ORR of 93.3% in squamous cancer, 40% in non-squamous cancer, and a DCR of 100% (90). The drug safety was good, with an overall TRAE incidence of 61.4%, including 34.1% of ≥ grade 3 TRAEs (90). The above results indicate that the first-line treatment of PD-L1-negative advanced NSCLC with cadonilimab in combination with chemotherapy shows an auspicious therapeutic effect, especially in patients with squamous carcinoma.

MEDI5752 is a 1 + 1 form of an asymmetric bispecific antibody that preferentially binds CTLA-4 from PD-1^+^ T cells by reducing the affinity to the CTLA-4 (80). In a head-to-head comparative trial with Keytruda, patients treated with carboplatin plus pemetrexed plus MEDI5752 had better DOR, PFS, and OS than those treated with carboplatin plus pemetrexed plus Keytruda (mDOR: 20.5% vs. 9.9%, mPFS: 15.1 vs. 8.9 months, mDOR: NR vs. 16.5 months) (91). In addition, bsAbs based on PD-1 and CTLA-4 targeting include XmAb20717, SI-B003, and MGD019, some of which have published preliminary clinical data (**Table 1**). These bispecific antibodies, although not aligned with AK104 as well as MEDI5752 in terms of the specific protein sequences and format, are all designed to target both PD-1 and CTLA-4 and to bind PD-1/CTLA-4 double-positive cells to reduce CTLA-4 toxicity preferentially (124–126).

**Table 1:** Clinical results of bsAbs in lung cancer.

bsAb	Targets	INN	Sponsor	Format	Phase	Pts characteristics and intervention	Key results	ref
JNJ-61186372	EGFR cMET	Amivantamab	Janssen	Duobody (1 + 1)	approved (American)	Pts with *EGFR* Exon20ins advanced NSCLC who progressed after platinum-based chemo; Amiv mono; N=81;	ORR: 40%; mDOR: 11.1 mos.; mPFS: 8.3 mos.; mOS: 22.8 mos; TRAEs: 99%; Grade 3^+^ TRAEs: 35%;	(39)
						Pts with *EGFR* Exon20ins advanced NSCLC who had not received previous systemic therapy; Amiv + Chemo, N=151; Chem, N=155;	mPFS: 11.4 mos. vs. 6.7 mos.; PFS reported at 18 mos.: 31% vs. 3%; mOS: NR vs. 24.4 mos. (HR=0.67, 95% CI, 0.42–1.09); TRAEs: 100% vs. 98% Grade 3^+^ TRAEs: 75% vs. 54% (Amiv + Chem vs. Chemo)	(42)
						Pts with treatment-naïve, *EGFR-*mutated (Ex19del or L858R) locally advanced or metastatic NSCLC; Amiv + Lazertinib, N=429; Osimertinib, N=429;	ORR: 86% vs. 85%; mDoR: 25.8 mos.vs. 16.8 mos.; mPFS: 23.7 mos. vs. 16.6 mos. (HR=0.70, 95% CI, 0.58-0.85); Grade 3^+^ TRAEs: 75% vs. 43% (Amiv + Lazertinib vs. osimertinib)	(44) (45)
						Pts with relapsed or refractory NSCLC with *MET* exon 14 skipping mutation; Amiv mono; N=97;	ORR: 33%; mDoR: 11.2 mos.; CBR: 69%; Grade 3^+^ TRAEs: 42%;	(46)
						Pts with *EGFR*-mutated advanced NSCLC who progressed after osimertinib and platinum-based chem; Amiv subcutaneous combined with Lazertinib, N=206; Amiv intravenous combined with Lazertinib, N=212;	ORR: 30% vs. 33%; mPFS: 6.1 mos. vs. 4.3 mos.; mDoR:11.2 vs 8.3 mos.; OS: significantly longer (HR 0.62, 95% CI, 0.42- 0.92); IRRs: 13% vs. 66%; VTE: 9% vs. 14%;	(47)
EMB-01	EGFR cMET	Bafisontamab	EpimAb	FIT-Ig (2 + 2)	II	Pts with advanced solid tumors; EMB-01 mono; N=38;	ORR: 5.3%; SD: 36.8%; DCR: 42.1%; Grade 3^+^ TRAEs: 8.3% (rash); 1.7%(others);	(48)
MCLA-129	EGFR cMET	/	Merus	Biclonics (1 + 1)	II	Pts with relapsed or refractory NSCLC; MCLA-129 mono; *MET*ex14 mutation, N=23; *EGFR* Exon20ins, N=63; sensitized *EGFR*-mutated, N=55;	ORR: 43.5%; 28.6%; 21.8%; DCR: 95.7%; 84.1%; 69.1%; mDoR: 6.3 mos.; 7.2 mos.; 9.8 mos.; Grade 3^+^ TRAEs: 51.6%;	(50)
						Pts with advanced/metastatic *EGFR*mut NSCLC who were treatment-naïve or progressed on osimertinib; MCLA-129 + Osimertinib; treatment-naïve, N=16; progressed on Osimertinib, N=34;	ORR: 75.0%; 35.3%; DCR: 93.8%; 73.5%; Grade 3^+^ TRAEs: 23%; 38%;	(51)
MCLA-128	HER2 HER3	Zenocutuzumab	Merus	Biclonics (1 + 1)	II	Pts with relapsed or refractory advanced NRG1^+^ NSCLC; MCLA-128 mono; N=64;	ORR: 34%; mDoR: 12.9 mos.; Grade 3^+^ TRAEs: <4%;	(66)
SI-B001	EGFR HER3	Izalontamab	Baili	IgG1-scFv_2_ (2 + 2)	III	Patients with locally advanced or metastatic *EGFR/ALK* wild-type NSCLC who had failed first-line anti-PD-1/L1 therapy; SI-B001+ PBC/docetaxel; N=48;	ORR: 31.3%; DCR: 77.1%; Grade 3^+^ TRAEs: 17% (myelosuppression); 15% (decreased neutrophil count); 12% (decreased white blood cell count);	(70)
AK104	PD-1 CTLA-4	Cadonilimab	Akeso	Tetrabody (2 + 2)	approved (China)	Pts who had failed previous platinum-based doublet chemo and were immunotherapy naïve; AK104 mono; N=30;	ORR: 10%; DCR: 40%; mOS: 19.6 mos.; Grade 3^+^ TRAEs: 11.3%;	(92)
						Pts with advanced NSCLC; AK104 + anlotinib; treatment naïve, N=17; anti-PD-1/L1 resistant, N=6;	ORR: 70.6%; 16.7%; DCR: 94.1%; 100%; Grade 3^+^ TRAEs: 14.3%; 5.9%;	(93)
						Treatment naive Pts with advanced NSCLC; AK104 + anlotinib; N=69;	ORR: 53.6%; DCR: 92.8; mDoR: NR; Grade 3^+^ TRAEs: 49.3%;	(84)
						Pts with histologically or cytologically confirmed stage IIIB/IIIC or IV NSCLC without sensitizing EGFR/ALK/ROS1 mutations must had progressed during or after a PD-1/L1 inhibitor and a platinum-based chemotherapy; AK104 + anlotinib + docetaxel; N=33;	ORR: 30.3%; DCR: 94.0%; mPFS: 6.5 mos.; Grade 3^+^ TRAEs: 17.4%;	(85)
MEDI5752	PD-1 CTLA-4	Volrustomig	AstraZeneca	DuetMab (1 + 1)	III	Pts with NSCLC who were treatment-naïve; carboplatin/pemetrexed + MEDI5752, N=20; carboplatin/pemetrexed + pem, N=21;	ORR: 50%; 47.6%; ORR in PD-L1<1%: 55.6%; 30.0%; mPFS: 15.1 mos.; 8.9 mos.; mPFS in PD-L1<1%: 13.4 mos.; 9.0 mos.; mOS: NR; 16.5 mos.; Grade 3^+^ TRAEs: 32%, TEAE-D/C: 20%	(91)
SI-B003	PD-1 CTLA-4	/	Baili	IgG1-scFv_2_ (2 + 2)	I	Pts with recurrent or metastatic solid tumors who had failed standard therapy; SI-B003 mono; N=56)	ORR: 16.1%; DCR: 50.0%;	(94)
KN046	PD-L1 CTLA-4	Erfonrilimab	Alphamab	VHH-Fc (2 + 2)	III	Pts with *EGFR* sensitizing mutation (Ex19del or L858R), and failed from prior EGFR-TKI(s) without platinum-based chemo; KN046 + Pemetrexed+ carboplatin AUC5; N=26;	ORR: 26.9%; DCR: 84.6%; CB: 38.5%; mPFS: 5.5 mos.; mOS: 20.2 mos.; Grade 3^+^ TRAEs: 19.2% (infusion reaction); 15.4% (decreased platelet count); 11.5% (anemia);	(95)
						Pts with advanced NSCLC; KN046+ chemo; non-squamous NSCLC, N=51; squamous NSCLC, N=36;	ORR: 43.1%; 52.9% CBR: 50%; 61.1%; mDOR: 9.1 mos.; 7.3 mos.; mPFS: 5.8 mos.; 5.7 mos.; mOS: 27.2 mos.; 26.6 mos.; Grade 3^+^ TRAEs: 66.7%;	(96)
						Pts had NSCLC that had progressed after ICI(s) and platinum-based chemotherapy, excluding *EGFR* mutation and/or *ALK* translocation; KN046 mono; N=31;	ORR: 3.2%; DCR: 38.7%; mPFS: 2.8 mos.; mOS: 13.3 mos.; Grade 3^+^ TRAEs: 9.7% (anemia); 3.2% (febrile neutropenia); 3.2% (fatigue);	(97)
						Pts with stage IIIB-IV NSCLC and without *EGFR* activating mutation and *ALK* rearrangement; KN046 +axitinib; treatment-naive and PD-L1 expression ≥1%, N=44; treatment-naive and PD-L1 expression ≥50%, N=15; progressed on CPIs, N=32);	ORR: 56.8%; 73.3%; 9.4%; DC: 90.9%; 93.3%; 81.3%; mDOR: 13.2 mos.; NE; 7.4 mos.; mPFS: 8.3 mos.; 12.4 mos.; 5.6 mos.; Grade 3^+^ TRAEs: 58.5%; 59.4%; 58.8%;	(98)
IBI318/LY3434172	PD-1 PD-L1	/	Innovent/Lilly	IgG like (1 + 1)	II	Pts with advanced NSCLC; IBI318/LY3434172 mono;IO-failed NSCLC, N= 10; immunotherapy-naïve NSCLC pts with a PD-L1 TPS of 1–49%, N=8; treatment-naïve NSCLC pts with a PD-L1 TPS ≥ 50%, N=11;	ORR: 0%; 12.5%; 45.5%; DCR: 30%; 50%; 81.8%; Grade 3^+^ TRAEs: 8.2%;	(99)
AZD2936	PD-1 TIGIT	Rilvegostomig	AstraZeneca	DuetMab (1 + 1)	III	Pts with advanced NSCLC who had prior CPIs treatment and a PD-L1 tumor proportion score ≥1%; AZD2936 mono; PD-L1 TPS=1-49%, 750mg, N=31; PD-L1 TPS≥50%, 750mg, N=34; PD-L1 TPS≥50%, 1500mg, N=30;	ORR:29%; 61.8%; 36.7%; DCR:64.5%; 88.3%; 66.7%; mDoR (all confirmed responders): 10.5 mos.; discontinued due to TRAEs: 4.2%; Grade 3^+^ TRAEs: 10.5%	(100)
PM1022	PD-L1 TIGIT	/	Biotheus	IgG-VHH (2 + 2)		Pts with advanced solid tumors; PM1022 mono; N=15;	ORR: 7.1%; DCR: 35.7%; TRAEs: 53.3%;	(101)
MDG103	PD-1 LAG-3	Tebotelimab	MacroGenics	DART-Fc (2 + 2)	III	Pts with advanced or metastatic NSCLC; MDG103 mono; CPIs naive, N=14; post-CPI, N=15;	ORR: 14.3%; 0; DCR: 64.3%; 53.3%; Grade 3 + TRAEs: 50.5%;	(102)
AK112	PD-1 VEGF-A	Ivonescimab	Akeso/Summit	Tetrabody (2 + 2)	approved (China)	NSCLC pts with *EGFR* mutations who had failed prior EGFR-TKIs therapies; Ivon + chemo, N=161; placebo + chemo, N=161;	ORR: 50.6% vs. 35.4%; mPFS: 7.06 mos. vs. 4.80 mos.; mDOR: 6.6 mos. vs. 4.2 mos.; PFS: significantly improved (HR 0.46, 0.34-0.62); OS: significantly improved(HR 0.8, 95% CI, 0.59-1.08) Grade 3^+^ TEAEs: 61.5% vs. 49.1%; (Ivon + chemo vs. placebo + chemo)	(103)
						Pts with previous untreated stage IIIB to IV advanced NSCLC (*EGFR/ALK* wild-type and PD-L1≥1%); Ivon, N=198; Pemb, N=200;	ORR: 50.0% vs. 38.5%; DCR: 89.9% vs. 70.5%; mPFS: 11.14 mos. vs. 5.82 mos.; (HR 0.51; 0.38-0.69) TEAEs: 89.8% vs. 81.9% Grade 3^+^ TEAEs: 29.4% vs. 15.6%; (Ivonescimab vs Pembrolizumab)	(104)
PM8002	PD-L1 VEGF-A	/	Biotheus/BioNTech	IgG-VHH (2 + 2)	III	Pts with advanced NSCLC; PM8002 mono; Treatment-naïve no-sq-NSCLC with *EGFR/ALK* wild-type and PD-L1^+^, N=17; EGRF-TKI treated no-sq-NSCLC, N=36; IO and PBC treated NSCLC with *EGFR/ALK* wild-type, N=8;	ORR: 47.1%; 19.4%; 12.5%; mPFS: 10.9 mos.; 4.9 mos.; 6.7 mos.; 6 mos. PFS: 82.4%; 43.8%; 62.5%; Grade 3^+^ TEAEs: 18%;	(105)
						Pts with advanced SCLC who failed first-line platinum-based chemo with or without CPIs therapy; PM8002 mono; N=22;	ORR: 72.7%; DCR: 81.8%; mPFS: 5.5 mos.; Grade 3^+^ TEAEs: 18%	(106)
IMM2510	PD-L1 VEGFs	/	ImmuneOnco/Instil Bio	IgG-fusion protein (2 + 2)	Ib/II	Pts with advanced solid tumors; IMM2510 mono; N=25;	ORR: 12% (3/25); DOR: 40% (10/25); Grade 3+ TEAEs: 33.3%;	(107)
SHR-1701	PD-L1 TGF-β	Retlirafusp alfa	Suzhou Suncadia	IgG-fusion protein (2 + 2)	III	Pts with advanced/metastatic NSCLC; SHR-1701 mono; Treatment-naïve NSCLC with PD-L1^+^, N=57; EGFR TKIs treated or no standard EGFR TKIs were available NSCLC, N=41; CPIs treated pts who had received up to 3 previous lines of treatments, N=33;	ORR: 36.8%; 19.5%; 9.1% DCR: 66.7%; 46.3%; 54.5% mPFS: 5.3 mos.; 1.4 mos.; 2.1 mos.; mOS: 24.2 mos.; 14.4 mos.; 16.1 mos.; Grade 3^+^ TEAEs: 22.9%;	(108)
						Pts with unresectable stage III NSCLC; SHR-1701 plus chem followed by surgery or radiotherapy, and then consolidation SHR-1701; N=107;	post-induction ORR: 58%; 18-month EFS: 56.6%; Pts underwent surgery: 25%; Pts achieved R0 resection: 25%; mPR: 12%; cPR: 6.5%;	(109)
AMG757	DLL3 CD3	Tarlatamab	Amgen	HEL-BiTE (1 + 1)	approved (American)	Pts with advanced SCLC previously treated with two or more lines of therapy; Tarl mono; 10mg-group, N=100; 100mg-group, N=88;	ORR:40%; 32%; mPFS: 4.9 mos; 3.9 mos.; mOS: 14.3 mos.; NE; CRS: 51%; 61% Grade 3^+^ TEAEs: 59.4%; 64%; Fatal: 5.3%; 6%;	(110)
						Pts with previously treated SCLC; Tarl mono; Tarl≥10mg, N=152; 10 mg Tarl Q2W, n=17; )	ORR:25%; 35.3%; mDOR: 11.2 mos.; 19.4 mos.; mOS: 17.5 mos.; 20.3 mos.; Intracranial DCR (all): 87.5% (14/16);	(111)
BI-764532 / OBT-620	DLL3 CD3	/	Boehringer Ingelheim/Oxford Bio Therapeutics	IgG like (1 + 1)	I	Pts with locally advanced/metastatic DLL3^+^ (confirmed centrally) solid tumors; BI-764532 mono; SCLC, N=39; epNEC, N=27; LCNEC, N=5; ALL, N=71;	PR: 26%; 19%; 60%; 25%; DCR: 51%; 44%; 100%; 52%; Grade 3^+^ TEAEs: 27% Discontinued due to TRAEs: 4%;	(112)
HPN328/MK6070	DLL3 CD3 HSA	/	Harpoon/Merck	TriTAC (1 + 1+1)	I/II	Pts with relapsed/refractory, metastatic SCLC and other NEN associated with DLL3 expression; HPN328 mon; SCLC, N=28; other NEN, N=13;	ORR: 39%; 46%; DCR:71%; 46%; Grade 3^+^ TEAEs: 26%; discontinued due to TRAEs: 4%; death due to TRAEs: 2%;	(113)
						Pts with relapsed/refractory, metastatic SCLC; HPN328 mono; Brain metastases, N=28; No brain metastases, N=21;	ORR:37%; 19%; DCR: 78%; 48%;	(114)
JANX008	EGFR CD3	/	Janux	TRACTr (1 + 1+1)	I	Pts with advanced or metastatic solid tumors known to express high levels of the EGFR target JANX008 mono; N=11;	one Pt with NSCLC had a confirmed PR with 100% reduction of the target lung lesion and elimination of liver metastasis; Grade 1 CRS in 2 Pts;	(115)
GEN1046	PD-L1 4-1BB	Acasunlimab	Genmab	Duobody (1 + 1)	III	Pts with advanced solid tumors; GEN1046 mono; N=61;	DCR: 65.6%; Grade 3^+^ TEAEs: 21.3%; DLT: 9.8%	(116)
						Pts with PD-L1^+^ metastatic NSCLC who had disease progression following one or more prior lines of anti-PD-1/L1-containing treatment; arm A, GEN1046 100 mg Q3W x 2 cycles then 500 mg Q6W, N=16; arm B, GEN1046 100 mg + pemb 200 mg Q3W, N=22; arm C, GEN1046 100 mg + pemb 400 mg Q6W, N=24;	ORR: 12.5%; 18.2%; 16.7%; DCR: 50%; 59.1%; 75%; mDOR: 2.0 mos.; 5.2mos.; NR mos.; mOS: 5.5 mos.; 8.6 mos.; 17.5 mos.; Grade 3^+^ TEAEs: liver reated events (9.1%; 16.7%; 12.2%); anemia (4.5%; 2.4%; 0%);	(117)
FS222	PD-L1 4-1BB	/	F-star	mAb2 (2 + 2)	I	Pts with pretreated advanced solid tumors; FS222 mono; N=90;	ORR: 15.7% (include NSCLC); Grade 3^+^ TEAEs (≥10% of pts): AST (13.3%); ALT (11.1%);	(118)
AFM24	EGFR CD16	/	Affimed	IgG1-scFv_2_ (2 + 2)	III	Pts with *EGFR* mutant NSCLC, relapsed or refractory to ≥1 prior lines of therapy; AFM24 mono, N=10;	DCR: 50%; Grade 3^+^ TEAEs: 40%; Grade 5 pneumonitis: 1/10	(119)
						Pts with advanced or metastatic *EGFR*-WT NSCLC who progressed on ≥1 prior line of therapy, including at least a platinum doublet and a CPI; AFM24+atezolizumab, n=15;	ORR: 26.7%; DCR: 73.3% Grade 3+ TEAEs: 13.3%	(120)
IBI363	PD-1 IL-2	/	Innovent	IgG-fusion protein (1 + 1)	II	Pts with advanced non-small cell lung cancer IBI363 mono, N=79;	ORR: 24.1%; DCR: 68.4%; Grade 3^+^ TEAEs: 19.1%; discontinued due to TRAEs: 4.5%; death due to TRAEs: 1.1%;	(22)
PF-07209960	PD-1 IL-15	/	Pfizer	IgG-fusion protein (2 + 1)	I	Pts with advanced or metastatic solid tumors, N=29;	ORR: 6.9%; DCR: 48.3% Grade 3^+^ TEAEs: 778.4%;	(121)
SAR44587/KD055	PD-1 IL-15/IL15-Rα	/	Sanofi	IgG-fusion protein (2 + 1)	I	/	/	(122)
IAP0971	PD-1 IL-15/IL15-Rα		SunHo Bio	IgG-fusion protein (2 + 1)	I	/	/	(123)

bsAb, bispecific antibody; INN, international nonproprietary name; pts, patients; ref, reference; EGFR, epidermal growth factor receptor; cMET, c-mesenchymal-epithelial transition factor; NSCLC, no small cell lung cancer; chemo, chemotherapy; amiv, amivantamab; mono, monotherapy; N, number of efficacy population; ORR, objective response rate; CI, confidence interval; mDOR, median duration of response; mos., months; NR, not reached; mPFS, median progression-free survival; mOS, median overall survival; TRAE, treatment-related adverse events; CBR, clinical benefit ratio; VTE, venous thromboembolism; SD, stable disease; DCR, disease control rate; NRG1, neuregulin 1; PD-1, programed cell death protein 1; PD-L1, programed death-ligand 1; PBC, platinum-based chemotherapy; CTLA-4, cytotoxic T lymphocyte-associated antigen-4; HER2, human epidermal growth factor receptor-2; HER3, human epidermal growth factor receptor-3; pemb, pembrolizumab; ICI, immune checkpoint inhibitor; TIGIT, T cell immunoreceptor with Ig and ITIM domains; CPIs, checkpoint inhibitors; LAG3, lymphocyte activation gene-3; VEGF-A, vascular endothelial growth factor A; Ivon, Ivonescimab; no-sq-NSCLC, non-squamous NSCLC; TGF-β, transforming growth factor-β; DLL3, delta-like protein 3; SCLC, small cell lung cancer; Tarl, tarlatamab; Q2W, once every two weeks; CRS, cytokine-release syndrome; epNEC, extrapulmonary neuroendocrine carcinoma; LCNEC, large cell neuroendocrine carcinoma; NEN, neuroendocrine neoplasms; PR, partial response.

Erfonrilimab (KN046) is a tetravalent symmetric bsAb targeting PD-L1 and CTLA-4, consisting of two identical chains, each consisting of a PD-L1 single-domain antibody, a CTLA-4 single-domain antibody, and a Fc domain (87). By design, the antibody has a higher affinity for PD-L1 and, therefore, can preferentially target the tumor microenvironment with high PD-L1 expression to reduce toxicities (96). At the same time, the antibody retains the function of Fc to remove CTLA-4-expressing Treg in the tumor microenvironment (127). In the study named KN046-201 (NCT03838848), a total of 26 advanced NSCLC patients with EGFR sensitivity mutation who had failed EGFR-TKI(s) and without platinum-based chemotherapy were enrolled and were given KN046 in combination with pemetrexed and carboplatin as a second-line treatment (95). The reported ORR was 26.9%, mPFS was 5.5 months, and mOS was 20.2 months (95). And 57.7% of the patients experienced grade 3 or higher TRAEs (95). In another phase II study evaluating KN046 in combination with chemotherapy for the first-line treatment of patients with metastatic NSCLC (NCT04054531), the ORR was 46.0%, with a mPFS of 5.8 months and a mOS of 26.6 months (96). In addition, for patients with metastatic NSCLC who had failed previous immunotherapy and platinum-based chemotherapy, the mOS of patients given KN046 was up to 13.3 months (97). In a study evaluating KN046 in combination with axitinib in advanced NSCLC (NCT05420220), for previously untreated patients with PD-L1 TPS ≥1% and patients treated with CPIs, the ORR after receiving the combination therapy was 56.8% and 9.4% respectively, and the DCR was 90.9% and 81.3%, showing promising efficacy (98).

#### 4.2.2 PD-1 × PD-L1

IBI318/LY3434172 is an IgG-like dual antibody targeting PD-L1 and PD-1, and preclinical data suggest that it has significant tumor-suppressive effects and is superior to equivalent doses of monoclonal antibodies, as well as the combination of PD-1 and PD-L1 monoclonal antibodies (128). Preliminary phase Ib clinical (NCT03875157) data suggests that this drug has significant efficacy in immunotherapy-naïve NSCLC patients who had failed or were intolerant to first-line chemotherapy. Its ORR in patients with PD-L1 scores of 1-49% and ≥50% was 12.5% (1/8) and 45.5% (5/11), respectively, and its DCR was 50% (4/8) and 81.8% (9/11), respectively (99).

#### 4.2.3 PD-1/(L)1 × TIGIT

T cell immunoglobulin and ITIM domain (TIGIT) is an immune checkpoint that has recently attracted attention. TIGIT interacts with CD155 (poliovirus receptor, PVR, or NECL-5) on the surface of antigen-presenting cells or tumor cells and inhibits the anti-tumor response of T cells and NK cells (129). TIGIT is expressed in various lung cancers, including NSCLC, and overexpression of TIGIT/CD155 is an unfavorable prognostic factor in lung adenocarcinoma (130). TIGIT is generally co-expressed with immunosuppressive molecules, such as PD-1, on various T cells, and both inhibit CD8^+^ T cell activity through different mechanisms (131, 132). TIGIT blockade is a promising immunotherapy in terms of molecular mechanisms. However, vibostolimab and tiragolumab, two monoclonal antibody drugs targeting TITGI, are ineffective as monotherapy (133, 134). However, tiragolumab in combination with atezolizumab had an overall ORR of 37% and an ORR of 66% in the PD-L1 TPS>50% subgroup, which exceeded atezolizumab monotherapy (21% and 24%, respectively) (135). Therefore, developing bsAb targeting PD-1 and TIGIT is also interesting to researchers.

Rilvegostomig (AZD2936) is an asymmetric dual antibody targeting PD-1 and TIGIT. The initial efficacy of rilvegostomig in patients with advanced NSCLC treated with CPIs has been published (NCT04995523). Patients with PD-L1 TPS ≥50% treated with rilvegostomig 750mg had an ORR of 61.8% and a DCR of 88.3%, and the drug had a favorable safety profile, with a grade 3 or higher adverse events rate of 10.5% (100). The company developing the drug is currently conducting a head-to-head clinical trial with Keytruda, which shows the drug developer’s confidence in this drug.

PM1022 is a bispecific antibody targeting PD-L1 and TIGIT, with VHH targeting PD-L1 fused to the C-terminus of the anti-TIGIT antibody. The antibody is currently undergoing a dose extension clinical study (NCT05867771), and preliminary results show an ORR of 7.1%, a DCR of 35.7%, and an overall TRAE rate of 53.3% in treated patients (101). One of the NSCLC patients had received prior treatment, including chemotherapy and anti-PD-1 therapy, and had a 56.8% reduction in lesion size (101).

#### 4.2.4 PD-1 × LAG-3

Lymphocyte activation gene 3 (LAG-3), the third-generation immune checkpoint receptor, is highly up-regulated on exhausted T cells in the tumor microenvironment (136). The binding of LAG-3 to its classical ligand, the major histocompatibility complex-II, leads to the down-regulation of T cell activation, proliferation, and cytokine production, ultimately causing T cell dysfunction (137). Immunohistochemistry analysis revealed that LAG-3 is expressed in various cancer tissues, with 90% of NSCLC and 52% of SCLC samples having detectable positive LAG-3 expression (102). In NCSLC, co-expression of PD-1 and LAG-3 was detectable in 59% of samples (102). Moreover, it was found that LAG-3 and PD-1 synergize on CD8^+^ T cells to drive T cell exhaustion (138). Currently, the FDA approved Opdualag (consisting of a fixed-dose combination of relatlimab and nivolumab) for treating unresectable or metastatic melanoma in 2022 (139). In addition, it was shown that compared to nivolumab combination chemotherapy, Opdualag combination chemotherapy improved ORR (53.2% vs. 40.8%), prolonged mPFS (9.8 vs. 6.1 months), and had an HR of 0.63 in this group of NSCLC patients with PDL1 ≥ 1%; and, for non-squamous NSCLC (non-sq-NSCLC) patients with PD-L1 TPS≥1%, the ORR improved to 58%, the mPFS increased to 11.6 months, and the HR was further reduced to 0.55 (140). The above study demonstrated that dual immune checkpoint therapy with LAG-3 and PD-1 has a better prospect in lung cancer treatment.

MGD103 (Tebotelimab) is a tetravalent DART-Fc (IgG4κ) fusion protein that blocks PD-1 and LAG-3. *In vitro* studies have shown that this bsAbs is significantly more potent than the combination of nivolumab and relatlimab in stimulating IFN-γ secretion (102). Tebotelimab had an ORR of 14.3% and a DCR of 64.3% in NSCLC patients who did not receive CPIs; however, no remission was observed in NSCLC patients who had previously received CPIs (NCT03219268) (102). Interestingly, there was a correlation between objective response to tebotelimab and LAG-3 expression (P < 0.05), but no statistical association between PD-1 and clinical response was observed (102).

In addition to the aforementioned immunomodulatory targets with more basic and clinical studies, some new immune checkpoint molecules (e.g., OX40, TIM3) have also attracted the attention of researchers in the past decade (141, 142). Moreover, there is much clinical or preclinical evidence that these new immune checkpoints synergize with anti-PD-1/L1 and/or anti-CTLA-4 monoclonal antibodies (143). Therefore, it is worth waiting for the final clinical outcome based on these newly discovered immune checkpoints combined with PD-1/L1 or anti-CTLA -4 to form a bsAbs.

### 4.3 Dual inhibition of tumor microenvironment and immune checkpoints

A tumor is a heterogeneous complex of tumor cells, various non-malignant cells (e.g., immune cells, stromal cells, endothelial cells, cancer-associated fibroblasts), and various non-cellular components (e.g., vascularised extracellular matrix, exosomes, cytokines) (144). The tumor microenvironment (TME) influences tumor growth, metastatic spread, and response to therapy. It has become common knowledge that TME-mediated immunosuppression impairs beneficial responses.

#### 4.3.1 PD-1/L1 × VEGF

In NSCLC, high vascular endothelial growth factor (VEGFs) expression is associated with tumor recurrence, low survival, metastasis, and death in patients (145, 146). Overexpression of VEGF induces a reduction in the expression of endothelial cell adhesion molecules, which severely impairs T-cell homing and reduces the number of T lymphocytes entering the TME (147). It has been shown that VEGF-A also enhances the expression of PD-1 and other inhibitory checkpoints, such as CTLA-4, on the surface of T cells and inhibits the activity of CD8^+^ T cells, leading to a blockade of the effector function of T cells (148–150). Therefore, combining anti-angiogenic drugs with immunotherapy, which normalizes tumor vasculature through anti-angiogenic drugs and promotes the increase of tumor immune cells (e.g., tumor-infiltrating lymphocytes) in NSCLC, and utilizing immune checkpoint inhibitors which can unlock the functional inhibition of T cells by PD-1 and PD-L1, both act synergistically with each other thus showing better therapeutic effects within solid tumors (151–153). In the IMpower-150 trial, researchers tested atezolizumab plus bevacizumab plus chemotherapy as a first-line treatment for non-squamous NSCLC (non-sq-NSCLC), with an ORR and PFS of 63.5% and 8.3 months, respectively (154). Still, the incidence of adverse events in grades 3 or higher was as high as 58.5% (154). The FDA has approved atezolizumab, bevacizumab, and chemotherapy as first-line treatment for advanced non-squamous NSCLC (155).

AK112 (Ivonescimab) is a humanized anti-PD-1/VEGF-A bispecific monoclonal antibody in which an anti-PD-1 scFv fused to the end of each of the two heavy chains of the anti-VEGF-A antibody (bevacizumab) to form a tetravalent bis-antibody. This bispecific antibody can accumulate in the tumor microenvironment, effectively blocking both the PD-1 and VEGF pathways, inhibiting PD-1-mediated immunosuppression, and blocking tumor angiogenesis in the microenvironment (156, 157). In May 2024, China approved AK112 in combination with chemotherapy (pemetrexed + carboplatin) for patients with locally advanced or metastatic non-sq-NSCLC who are EGFR mutation-positive and have progressed after treatment with EGFR TKIs (158). The approval was based on a phase III clinical trial called HARMONi-A (NCT05184712), which showed that in patients resistant to EGFR TKIs, AK112 in combination with chemotherapy was sufficient to significantly prolong mPFS compared to placebo combination chemotherapy (7.06 months vs. 4.08 months; HR 0.46) and patients had a higher ORR (50.6% vs. 35.4%) (103). The primary results of HARMONi-2 (NCT05499390), a trial comparing the efficacy of ivonescimab with that of pembrolizumab, were also presented. It showed that in patients with previously untreated stage IIIB to IV advanced NSCLC who were *EGFR/ALK* wild-type and with PD-L1 TPS≥1%, AK112 significantly prolonged their mPFS compared to pembrolizumab (11.14 vs. 5.82 months; HR, 0.51; p<0.0001), increasing their ORR (50.0% vs. 38.5%) and DCR (89.9% vs. 70.5%) (104). Although the rate of serious adverse events in patients treated with AK112 was slightly higher than with pembrolizumab (Grade 3^+^ TRAEs: 29.4% vs. 15.6%), the results of HARMONi-2 are still encouraging (104). It is expected that AK112 will become a potential clinical option in the first-line treatment of lung cancer.

PM8002 is a humanized anti-PD-L1/VEGF-A bispecific antibody with the variable region of a humanized anti-PD-L1 nanobody fused at the end of the two heavy chains of the anti-VEGF-A antibody. Preliminary published data (NCT05918445) showed that in untreated *EGFR/ALK* wild-type and PD-L1^+^ patients who received PM8002, the ORR is 47.1%, the mPFS is 10.9 months, and the incidence of grade ≥3 TRAEs was 18% (105). In addition, another study (ChiCTR2200059911) demonstrated the efficiency of PM8002 combined with paclitaxel for second-line treatment of SCLC. It was reported that the ORR was 72.7% (16/22), the DCR was 81.8% (18/22), and the mPFS was 5.5 months (106). Notably, the recommended drug for second-line treatment of SCLC is topotecan, with an ORR of 22% and a mDoR of 7.6 months (159). Therefore, further observing the subsequent clinical efficacy of PM8002 in SCLC is worthwhile.

IMM2510 is a bispecific antibody targeting PD-L1 and VEGFs that incorporates a VEGF receptor 1 domain 2 (VEGFR1-D2) at the C-terminus of each of the two heavy chains of the anti-PD-L1 antibody, forming a VEGF trap capable of binding a wide range of VEGF receptor ligands in addition to VEGF-A (160). IMM2510 is currently in the preliminary phase I clinical studies (NCT05972460). As of 21 December 2023, 33 patients with advanced solid tumors were treated with IMM2510 at nine doses (0.007-20.0 mg/kg) (107). Among the 25 patients whose conditions were evaluable, 3 patients had achieved confirmed partial response, and 7 patients had stable disease (107). Among the PR patients, one patient with sq-NSCLC (onco-driver gene negative, previous IO treatment failure) who was treated with 3 mg/kg IMM2510 achieving tumor shrinkage of 46% and still on the treatment with treatment duration over 20 months; one sq-NSCLC treated with 10 mg/kg showed a tumor shrinkage of about 32% and a treatment duration of 9.4 months (107). TRAEs occurred in 32 pts (97.0%), and most were grade 1 or 2. Grade ≥3 TRAEs occurred in 11 pts (33.3%), and no DLT occurred (107).

#### 4.3.2 PD-L1 × TGF-β

A central factor in tumor immune resistance is immunosuppressive cytokines in the tumor microenvironment, a major component of which is transforming growth factor β (TGF-β). It has been shown that TGF-β upregulates PD-L1 transcription in tumors (161). Besides, TGF-β-mediated T-cell rejection is one of the mechanisms by which tumors become resistant to anti-PD-L1 therapy (162). TGF-β also induces the release of PD-L1-containing exosomes by tumor cells, and PD-L1 exosomes in tumor regions hamper the effector activity of CD8^+^ T cells (163). In addition, there is a bidirectional interaction between TGF-β and tumor-area hypoxia, where hypoxia is considered a key inducer of TGF-β, and the activity of the latter further enhances tumor-area hypoxia (164). Hypoxia induces upregulation of PD-L1 expression on tumor cells and upregulation of PD-1 expression on immune cells (e.g., TAMs, DCs, Tregs) in the tumor microenvironment (165, 166). Aberrant TGF-β activity is associated with immunosuppressive TME, promoting progression and metastasis in NSCLC (167). Several companies have developed drug molecules with dual inhibitory effects on PD-1/L1 and TGF-β, among which M7824 and SHR1701 were developed earlier and have more preclinical and clinical data.

M7824 is a bifunctional fusion protein, a molecule that fuses a TGF-β receptor II (a TGF-β ‘trap’ which is capable of trapping all TGF-β) to the end of the constant region of avelumab via a flexible linker (168). M7824 has been noticed and given high expectations based on its phase I clinical (NCT02517398) results released in 2018. Among 40 patients with advanced NSCLC who relapsed after standard therapy treated with 1200 mg of M7824, the ORR was 40.7% for PD-L1-positive patients and 71.4% for patients with high PD-L1 expression (169). However, the subsequent publication of several clinical data has overshadowed M7824’s prospects. In the phase III clinical trial (NCT03631706) using either M7824 or pembrolizumab as first-line treatment for patients with advanced PD-L1 high-expression (TPS ≥80%) NSCLC, the results showed that first-line treatment with M7824 did not demonstrate superior efficacy compared to pembrolizumab and had a higher frequency of associated adverse events (170). Combined with unsatisfactory results from multiple other clinical trials, the outlook for M7824 is troubling, and clinical trials for the drug have now primarily been terminated.

SHR-1701 is also a bifunctional fusion protein that fuses an extracellular TGF-β receptor II structure to the C-terminus of a monoclonal antibody against PD-L1 (171). At the ESMO 2023 meeting, researchers preliminarily presented the results of SHR-1701 in 3 NSCLC clinical expansion cohorts (NCT03774979). For patients who had not received systemic chemotherapy and had a PD-L1 TPS ≥1%, ORR was 36.8%, DCR was 66.7%, and mOS was 24.2 months; for patients with an EGFR mutation who had failed prior treatment with standard EGFR TKIs or had no standard EGFR TKIs available, ORR was 19.5%, DCR was 46.3%, and mOS was 14.4 months; for patients who experienced disease progression after recent anti-PD-1/PD-L1 therapy and had received up to 3 lines of prior therapy ORR was 9.1%, DCR was 54.5%, mOS was 16.1 months (108). In this trial, 76.3% of patients treated with SHR-1701 experienced TRAEs, including 22.9% with grade ≥3 TRAEs (108). In addition, a trial (NCT04580498) was conducted to assess neoadjuvant SHR-1701 with or without chemotherapy, followed by surgery or radiotherapy, and then consolidation SHR-1701 in unresectable stage III NSCLC, and preliminary clinical results were recently published. Of the 97 evaluable patients treated with SHR-1701 plus chemotherapy, the postinduction ORR was 58%, and the 18-month event-free survival rate was 56.6% (109). Regarding safety, the rate of patients with grade ≥3 TRAEs was 75% in the SHR1701 plus chemotherapy arm (109). The drug is currently undergoing multiple multi-center phase III clinics, and there is much interest in the final outcome, especially as M7824 has been terminated from the clinic.

## 5 Immune cell engagement bispecific antibodies

### 5.1 T cell engagement

TCE is a typical application of bsAbs, with one targeting arm of most TCEs designed to bind specifically to selected tumor-associated antigen (TAA) on the surface of tumor cells and the other targeting arm designed to target the CD3ϵ chain in the TCR complex. Due to the signaling capacity of the CD3ϵ chain, TCEs can bypass the major histocompatibility complex restriction and, independently of the epitope specificity of the TCR, elicit T-cell activation and proliferation, as well as the subsequent release of transient inflammatory cytokines induced by the TCR and trigger tumor apoptosis via perforin and granzyme release (172). In addition, bsAbs targeting TAA and co-stimulatory receptors on T cells (e.g., 4-1BB, CD40, CD28, etc.) have a mechanism similar to that of a TCE.

#### 5.1.1 DLL3 × CD3

Delta-like ligand 3 (DLL3) is a TAA highly expressed on the surface of tumor cells in patients with SCLC, with high expression detected in approximately 85%-94% of SCLC patients (173, 174). Under normal conditions, DLL3 is mainly localized in the Golgi apparatus and cytoplasmic vesicles and is hardly expressed on the surface of normal cells (175). In contrast, in cells of SCLC and other high-grade neuroendocrine tumors, DLL3 is highly up-regulated and aberrantly expressed on the cell surface, leading to abnormal growth of neuroendocrine tumor cells (176). Thus, DLL3 has become a potential target for treating SCLC.

Tarlatamab (AMG757) is a DLL3-based TCE. According to published data from the clinical trial called Delphi -301 (NCT05060016), patients with SCLC who had received two or more systemic therapies benefited significantly from fortnightly intravenous treatment with Tarlatamab (110, 177). This study demonstrated a more significant benefit in patients treated with 10 mg of Tarlatamab compared to 100 mg, with an ORR of 40.0%, mPFS of 4.9 months, and mOS of 14.3 months (110, 177). The most common adverse events were cytokine release syndrome (in 51% of the patients in the 10-mg group), decreased appetite (29%), pyrexia (35%), constipation (27%), and anemia (26%) (110, 177). Grade 3 or higher adverse events occurred in 59% of the patients in the 10-mg group (110, 177). Based on the clinical data published in Delphi-301, the FDA granted tarlatamab accelerated approval in May 2024 for the treatment of extensive-stage SCLC, where disease progression occurs during or after platinum-based chemotherapy (178). In addition, newly published long-term follow-up data from Delphi-300 (NCT03319940) showed that the overall ORR for patients treated with more than 10 mg of tarlatamab was 25%, with an mDOR of 11.2 months (111). Patients with brain metastases also showed significant benefits after treatment with tarlatamab, with intracranial disease control occurring in 87.5% (111). Safety data were consistent with Delphi -301, with no new safety signals (111). TCEs in solid tumors have long been considered challenging (172), and tarlatamab is the first TCE therapy to be approved for treating a solid tumor, marking a critical step forward.

BI764532 is an IgG-like TCE that induces strictly DLL3-dependent tumor killing (179). BI764532 is currently in phase I clinical trial (NCT04429087), and preliminary clinical data have been published. Of 71 evaluable patients treated with no less than 90ug/kg of BI764532, 25% achieved partial response, with a DCR of 52%, and tumor shrinkage was observed in all patients (112). A total of 86% of patients experienced adverse events of any grade, with 37% experiencing grade 3^+^ TRAEs and four non-Asian patients discontinuing due to TRAEs (112).

PN328/MK6070 is a TCE targeting DLL3 and CD3. In addition, to prolong the half-life of this TCE as well as to maintain its most substantial direct T-cell killing ability, an anti-HSA single-domain antibody was fused in the middle of the DLL3 and CD3 targeting arms (see **Figure 1**) (180). It is currently enrolling participants with SCLC, neuroendocrine prostate cancer, and other high-grade neuroendocrine neoplasms in a phase I/II clinical trial (NCT04471727). The data showed that in the dose-optimized cohort (1-mg priming dose and a 12- or 24-mg target dose), patients with SCLC had an ORR of 39% and a DCR of 71% (113). In addition, the data showed that SCLC patients with brain metastases also responded well to HPN328 (ORR of 37% and DCR of 78%) (114). In terms of the safety of the drug, the incidence of TRAEs at any grade was 93%, with grade 3^+^ TRAEs occurring in 26%, with four patients discontinuing the drug due to TRAEs and two patients dying (113).

#### 5.1.2 EGFR × CD3

As mentioned earlier, EGFR is highly expressed in a variety of tumors. Meanwhile, EGFR regulates the development and homeostasis of regulated epithelial tissues in normal tissues and plays a key role in epithelial cell physiology (181). Therefore, a variety of drugs targeting EGFR have some skin toxicity (182, 183). In contrast, TCE-induced tumor cell killing is highly efficient, as TCE enables individual T cells to connect with multiple tumor cells, resulting in sequential killing (184). In addition, cytokines released from activated T cells can generate a cascade amplification effect to achieve a more excellent range of tumor cell killing (185). Therefore, TCEs targeting EGFR face more severe on-target off-tumor toxicity, resulting in a limited therapeutic window. However, suppose TCEs are engineered with a modified design to specifically recognize EGFR on tumor cells and bind no or less EGFR on normal tissues. In that case, they can significantly enhance the efficacy and expand the drug’s therapeutic window.

JANX008 is a prodrug form of TCE that targets EGFR and contains an EGFR-binding domain, a CD3-binding domain, and a HAS-binding domain that serves to extend the half-life of the molecule (see **Figure 1c**) (186). In addition, a mask is fused to each of the EGFR and CD3 binding domains through tumor protease cleavable linkers, and only when the molecule enters the tumor site and is recognized by the tumor protease can the peptide masks be released, ultimately generating the active molecules (186). The drug currently enrolls patients with advanced or metastatic cancers with high EGFR expression in a clinical phase Ia trial. It has been observed to achieve partial remission in one NSCLC subject, with a 100% reduction in targeted lung lesions and the elimination of liver metastases (115). Notably, JANX008 had a favorable safety profile, with grade 1 CRS observed in only 2 of 11 subjects at doses up to 1.25 mg (significantly higher than the expected maximum tolerated dose of the parental T-cell articulator) (115).

#### 5.1.3 PDL-1 × 4-1BB

4-1BB [also known as CD137 or TNF receptor superfamily member 9 (TNFRSF9)] is an inducible co-stimulatory receptor expressed on activated T cells and NK cells (187, 188). The agonism of 4-1BB avoids tumor-infiltrating lymphocyte exhaustion and enhances the antitumor activity of ICIs (189, 190). Although preclinical results have shown excellent efficacy of anti-4-1BB antibodies in different tumors, the development of 4-1BB-based monoclonal antibodies has successively failed either because of fatal side effects (urelumab) or because of limited efficacy (utomilumab) (191). Therefore, preserving the efficacy of anti-4-1BB antibodies while reducing their toxicity is a priority for subsequent development. One such strategy is the use of bsAb, which minimizes off-target tumor toxicity by designing the antibody to preferentially bind specifically to tumor cells and be enriched in the tumor microenvironment, and then to bind to 4-1BB and achieve activation of 4-1BB signaling. In this strategy, the most studied is the PD-L1/4-1BB bispecific antibody. Because PD-L1 is not only expressed on the surface of a wide range of cancer cells, a variety of host cells in the TME and lymph nodes, including dendritic cells, macrophages, fibroblasts, and T cells, also express PD-L1 to reduce antitumor immunity. In addition, PD-L1 blockade combined with 4-1BB agonistic antibodies has shown enhanced antitumor responses in preclinical cancer models (189, 190, 192). Thus, dual targeting of PD-L1 and 41BB by bispecific antibodies may permit tumor cell-dependent 4-1BB activation of T cells and allow optimal antitumor immunity.

GEN1046 (acasunlimab) is currently the fastest advancing bsAb targeting PD-L1 and 4-1BB. It is an IgG-like bsAb, with one arm targeting PD-L1 to block PD-1/PD-L1 inhibitory signals and another targeting 4-1BB to activate co-stimulatory signals in immune cells (116). A clinical trial (NCT05117242) evaluating acasunlimab monotherapy and acasunlimab in combination with pembrolizumab in metastatic NSCL patients who were resistant to anti-PD-1/L1 antibodies is currently underway, and preliminary results have been published. The data showed that patients treated with GEN1046 100 mg Q6W in combination with pembrolizumab 400 mg Q6W had a favorable treatment outcome (117). This subset of patients had an ORR of 16.7%, a DCR of 75%, a mOS of 17.5 months, and a 12-month OS rate of 69% (117). The most common adverse events (all grades; grade ≥3) in patients treated with the combination therapy included liver-related events (18.7%; 13.3%), fatigue (14.7%; 0%), malaise (13.3%; 0%), and diarrhea (12%; 0%) (117).

FS222 is a 2 + 2 tetravalent bsAbs constructed on a platform called mAb2. The antibody is based on a PD-L1 antibody, engineered to make its Fc recognize 4-1BB (193). The antibody is, therefore, similar in size to a conventional monoclonal antibody. Additionally, the antibody undergoes Fc mutation to reduce associated effects such as ADCC and CDC. FIH (NCT04740424) is an ongoing phase I clinical trial investigating the efficacy of FS222 in advanced solid tumors. Currently, interim results from the Q4W cohorts have been reported. The cohort had a total of 90 patients enrolled, with a median of 2 (1–7) regimens previously treated. At the cut-off date (05 Dec 2023), 20 (22.2%) patients were still on treatment, and objective remissions (CR, PR) were observed in patients with melanoma, NSCLC, ovarian, triple-negative breast, liposarcoma, and colon cancer with an ORR of 15.7% (118). The most common TRAEs grade ≥3 (≥10% of pts) were increased AST (13.3%) and ALT (11.1%) (118).

### 5.2 NK cell engagement

NK cells are innate immune cells with cytotoxicity. NKCEs have one targeting arm targeting tumor cell surface-specific antigens and the other targeting arm targeting activating receptors on the surface of NK cells, such as CD16a, NNKG2D (atural Killer Group 2 Member D), NKp30 (Natural Killer cell p30), etc., resulting in the formation of antigen-specific immune synapses between the NK cells and the tumor cells that which in turn triggers NK cell-mediated killing of tumor cells (194). NKCEs are a new exploration, and several drugs of this type are undergoing preclinical or clinical evaluation.

#### 5.2.1 EGFR × CD16a

AFM24 is a tetravalent bispecific antibody in which the anti-EGFR scFv is fused to the c-terminus of the anti-CD16a antibody via a connector, which mediates the killing of tumor cells by NK cells in an EGFR-dependent manner (195). Studies have demonstrated the preliminary efficacy of AMF24 in NSCLC patients with *EGFR* mutant NSCLC, relapsed or refractory to ≥1 prior lines of therapy. Of the 10 evaluable patients, 1 patient suffered a 45% reduction in tumor volume, and 4 had stable disease with a 50% tumor control rate (119). However, a high proportion of patients experienced TRAEs (13/14), with grade ≥3 TRAEs occurring in 4 patients and grade 5 pneumonitis occurring in 1 patient (13/14) (119). In NSCLC patients with *EGFR*-WT who relapsed after one or more first-line therapies, the combination of AFM24 and atezolizumab led to 1 complete response, 3 partial responses, and 7 cases of stable disease among the 15 evaluable patients (120). Moreover, this combination therapy was well tolerated. The main adverse events that occurred in the patients were infusion-related reactions (10/17) (120).

## 6 Immunocytokines

Cytokines are mediators and modulators of the innate and adaptive immune systems, and immunotherapy based on cytokines such as interferon and interleukin-2 (IL-2) has been used in the treatment of cancer as early as the end of the 20th century (196). However, the short half-life of cytokines results in the need for short periods of high-dose administration, which can lead to severe non-specific toxicity. In addition, the cytokine’s inhibitory or activating effect on immune cells is also related to the concentration and the environment of action. Therefore, engineering cytokines to preferentially target disease sites and to activate only specific lymphocyte types can increase the tolerability of cytokine therapy. The specific targeting of antibodies naturally makes them ideal ‘carriers’ for targeted delivery of therapeutic cytokines. In many mouse models, antibody-cytokine fusion proteins targeting tumor markers increase the selective accumulation of the corresponding cytokines at the tumor site (19, 20). Such antibody-cytokine fusion proteins, also known as immunecytokines, are another significant application of bispecific antibodies and have been called the next generation of cytokine products.

### 6.1 PD-1 × IL-2

High-dose recombinant IL-2 (Proleukin) was approved by the FDA for treating metastatic renal cell carcinoma in 1992, followed by metastatic melanoma in 1998 (197, 198). However, recombinant natural IL-2 is dose-limited (0.037 mg/kg admitted) and causes severe non-specific extravasation toxicity (197–199). In addition, IL-2 has two opposing functions: at low doses, IL-2 tends to stimulate Treg cells expressing high-affinity trimeric receptors (IL-2Rαβγ), resulting in immunosuppression (200, 201); at high doses, after saturation of the receptor on Treg cells, excess IL-2 also interacts with effector T cells via intermediate affinity receptors (IL-2Rβγ) binds to effector T and NK cells, promoting immune activation and anti-tumour responses (202). To improve the therapeutic index, researchers have engineered IL-2 through various strategies to promote a longer half-life of the modified drug and specific binding to IL2-Rβγ (197) without binding to IL2-Rα (203), expressed on Treg. However, these bias-modified drugs did not achieve better clinical results (204).

IBI363 is a PD-1/IL-2α-bias bispecific antibody fusion protein. Unlike the mainstream IL2-Rβγ-biased design, IBI363 fused an IL-2Rα-biased engineered modified IL-2 at the end of the anti-PD-1 antibody. This design was based on a preclinical correlative study, in which researchers found that newly activated tumor-specific CD8^+^ T cells expressed PD-1 while upregulating IL-2Rα and that the anti-tumor effect of anti-PD-1 was dependent on the activation of PD-1^+^ CD25^+^ CD8^+^ T cells via autocrine IL-2/IL2-Rα signaling (205). Thus, through specific guidance of PD-1, PD-1/IL-2α-bias can selectively stimulate and expand T cells expressing both PD-1 and IL-2Rα within the tumor, leading to more precise and effective targeting and activation of this T cell subpopulation. At WCLC 2024, researchers presented a phase I clinical study (NCT05460767) of IBI363 in advanced non-small cell lung cancer. The data showed an ORR of 24.1% and a DCR of 68.4% in 79 patients who received doses higher than 0.3 mg/kg and an ORR of 85.7% (6/7) in the 3 mg/kg dose group (22). The drug’s safety profile was good, with 87.6% of patients experiencing any grade of TRAEs and 19.1% of patients experiencing grade 3^+^ TRAEs (22). From the preliminary clinical data, it can be seen that IBI363 was administered at an unprecedented dose (3mg/kg) and demonstrated a favorable safety profile, breaking through the safety concerns of IL-2 therapy. Combined with its preliminary published efficacy data, the clinical results of subsequent treatments with IBI363 are promising.

### 6.2 PD-1 × IL-15

Interleukin 15 (IL-15) is an IL2-related cytokine. Both can bind IL-2Rβγ, but the high-affinity forms of both IL2 and IL15 receptors contain IL2-Rα (CD25) or IL-15Rα (CD215), respectively (206). IL-15 is produced by dendritic cells, macrophages, and stromal cells, and, like IL-2, IL-15 can stimulate T-cell proliferation, induce cytotoxic T lymphocyte production, and promote the maintenance of NK cells (207, 208). In addition, the unique role of IL-15 is to maintain NK cell and CD8^+^CD44^h^ memory T cell function to provide a long-term immune response to pathogen (208). Like IL-2, IL-15 therapy was initially limited by the short molecular half-life and toxicity associated with systemic immune activation (209). Anktiva (N-803) is a recombinant IL-15 superagonist protein complex consisting of a high-affinity IL-15 mutant and IL-15Rα fused to Fc (and thus with an extended half-life). 2024 The FDA approved Anktiva in combination with Bacillus Calmette-Guérin (BCG) for treating adult BCG-naïve patients with non-muscle invasive bladder cancer with carcinoma *in situ* with or without papillary tumors (210). Animal studies have demonstrated that the combination of N-803 and anti-PD-L1 antibody can activate NK and CD8⁺ T cells and induce the production of immunostimulatory cytokines, demonstrating significant efficacy in various models that do not respond or respond poorly to monotherapy (211). Clinical studies have demonstrated that patients with ≥2nd line NSCLC who failed CPI therapy treated with Anktiva in combination with CPI have an mOS of up to 11.4 months (212).

PF-07209960 is a cytokine fusion protein fused with mutated IL-15 at the end of one of the heavy chains of the anti-PD-1 antibody (213). The mutation in IL-15 is designed so that PF-07209960 does not bind to IL-15Rα and has a significantly reduced affinity for IL-2/15Rβγ. The molecule, therefore, explicitly delivers IL-15 to CD8+ T cells with high PD1 expression in tumors without binding to IL-15Rα-expressing cells and PD1-negative IL-15Rβγ-positive NK cells (213). Preclinical studies demonstrated that PF-07209960 could increase the number of CD8⁺ TILs within tumors specifically, had excellent anti-tumor activity, and significantly reduced adverse effects (213). Researchers recently published preliminary clinical data on PF-07209960. Of the 29 evaluable patients, two patients, both with microsatellite-stable colorectal cancer, were confirmed to be in partial remission, with an ORR of 6.9% and a DCR of 48.3% (121). Regarding safety, 97.3% of patients experienced one or more adverse events, of which 78.4% experienced grade 3 or higher TRAEs, with a serious adverse events rate of 70.3% (121).

SAR44587, or KD050, consists of an Fc-silenced high-affinity human anti-PD-1 antibody (IgG1 subtype) fused to a mutated IL-15/IL-15Rα sushi domain (122). Through its anti-PD1 portion, SAR44587 binds to PD-1-expressing T cells and NK cells and may result in the specific expansion and activation of CD8^+^ T cells and NK cells expressing PD-1 and IL-2/15Rβγ (214). In preclinical models of PD-1 resistance, SAR445877 had a stronger tumor suppressive effect compared to pembrolizumab, increasing the ratio of CD8^+^/CD4^+^ T cells in tumors and significantly increasing the percentage of effector memory CD8^+^ T cells in tumors (215). The drug is currently undergoing a clinical phase I study (NCT05584670), which is planned to enroll 240 adult patients with advanced unresectable or metastatic solid tumors to confirm the safety, tolerability, pharmacokinetics, pharmacodynamics, and antitumor activity (214).

IAP0971 is an immunocytokine that binds specifically to PD-1 and fused IL-15/IL-15Rα complex (123). Mechanistically, IAP0971 can deregulate the immunosuppression of the PD-1/PD-L1 axis while increasing the targeting of IL-15 to the tumor microenvironment and avoiding systemic non-specific activation; furthermore, IAP0971 has an IgG4-based structure, resulting in a weak effect such as ADCC and ADCP, and a long half-life (123). In preclinical studies, IAP0971 can stimulate the proliferation of CD8^+^ T cells and NKT cells, activate NK cells to kill tumor cells, and significantly inhibit tumor growth in mice at as low as 0.1 mg/kg without affecting their body weights (123). IAP0971 is currently in Phase I/IIa clinical trial (NCT05396391) to evaluate its safety, tolerability, and preliminary efficacy in patients with locally advanced or metastatic malignancies (216). Indications include a variety of malignancies, including lung cancer, cervical cancer, squamous cell carcinoma of the head and neck, hepatocellular carcinoma, and lymphoma.

## 7 Challenges and limitations

BsAbs show great potential in lung cancer therapy, and several key clinical advances are exciting. However, as described below, the therapeutic use of bsAbs in lung cancer faces many challenges.

### 7.1 Production challenges

Compared with monoclonal antibodies, the challenges facing the development and industrialization of bsABs are enormous. Unlike the standardized preparation process of monoclonal antibodies, the expression titer of bsABs is usually lower than that of monoclonal antibodies if the traditional process is directly followed (217), and they are prone to the formation of by-products such as aggregation and mismatch products (218–220). Although it is possible to increase the content of target products through various engineering modification strategies, bsAb-specific by-products are generally present at low levels in the cell culture supernatant of bsAb. It is necessary to rely on multiple purification strategies (e.g., a combination of affinity chromatography, ion-exchange chromatography, and size-exclusion chromatography) in order to obtain high purity of the target products, which significantly prolongs the development cycle and increases the cost of production (221).

### 7.2 Immunogenicity risk

Immunogenicity is one of the key challenges in the development of bsAbs. While some bispecific antibodies, such as amivantamab, have a very low incidence of anti-drug antibodies (ADAs), other bispecific antibodies show a high incidence of ADAs and neutralizing antibody positivity (222). These antibodies, which, due to drug immunogenicity, will directly affect the pharmacokinetics、pharmacodynamics, and safety of the drug (223). How to reduce the ADAs of a drug is a relatively complex issue, which needs to start from the initial molecular design (e.g., optimization of humanized modified epitopes), early *in vitro* immunogenicity assessment (based on in silico algorithms, and *in vitro* T cell-based assays), manufacturing process improvement (e.g., reduction of drug aggregation tendency, reduction of impurity residues), and clinical intervention (e.g., adjustment of drug immunogenicity), production process improvement (e.g., reducing drug aggregation tendency, reducing impurity residues), and clinical interventions (e.g., adjusting drug dosage, dosing regimen, and route of administration) are multifaceted and synergistic, which is complex and time-consuming (224).

### 7.3 Side effects

The adverse effects of bsAbs are closely related to their mechanism of action. Among them, cytokine release syndrome (CRS) is a common adverse reaction of TCE. For example, 49% of patients receiving AMG757 developed CRS, and 26% of them had severe symptoms (110). BsAbs based on a dual blocking mechanism show adverse reactions that are superimposed on the two targets (although they can be significantly lower than the parent antibody combination). For example, adverse reactions such as rash, pruritus, and diarrhea associated with EGFR and hypoproteinemia and peripheral edema associated with cMET have been observed with amivantamab (42).

Researchers are currently attempting various strategies to reduce the adverse effects of bsAbs and expand the therapeutic window. For example, by lowering the affinity of CD3 to increase the tissue distribution of bispecific antibodies in tumors and significantly reduce the level of cytokine release in normal tissues (225–227); and by restricting the activity of bsAbs in normal tissues through shielding techniques (186, 228); or by using tumor poxviruses as vectors to deliver dual antibodies to tumor sites in a targeted manner (229). However, drugs based on these strategies are currently in the preclinical or early clinical stage, and more clinical data are needed to analyze their safety and efficacy more correctly. Another strategy is to change the mode of administration. Currently, bsAbs are administered mainly by intravenous infusion, but data have shown that subcutaneous administration improves patient compliance and reduces the incidence of adverse events. For example, subcutaneous administration significantly reduced the incidence of infusion-related reactions and venous thromboembolic events with amivantamab compared with intravenous administration (47). Besides, some TCEs have also been shown through clinical studies to have a lower incidence of CRS with subcutaneous administration (230). However, subcutaneous administration is currently not widely used in solid tumors, and adequate subsequent validation is needed before widespread use. Intervention by the clinical therapist is the final barrier against adverse reactions. Real-time monitoring, pretreatment, and symptomatic management can enhance the patient experience (172, 231). However, it then requires clinicians to individualize the assessment of the patient and adjust the treatment regimen, which undoubtedly adds to the widespread use of the drug. Finally, it is worth noting that pneumonia is a particular concern in the lung cancer population, as lung cancer patients often have poor lung reserve due to current or past smoking history (232, 233). Drug-induced pneumonitis can, therefore, severely compromise their already poor lung reserve, which, in some cases, can be fatal.

### 7.4 Tumour heterogeneity and microenvironmental limitations

The complexity of lung cancer itself also poses a challenge for drug development. Lung cancer, as a very heterogeneous type of solid tumor, has differences in response to the same drug in different patients (as seen in **Table 1**). Thus, screening the most appropriate target population for bsAbs will be a significant challenge. In addition, the TME plays a central role in the genesis and progression of primary lung cancer, where cancer cells can reprogram tumor-infiltrating stromal cells, thereby promoting carcinogenesis (234, 235). In lung cancer, tumors can reprogram the lung microenvironment, which in turn promotes inflammation, angiogenesis, immunosuppression, and unresponsiveness to therapy, ultimately leading to lung metastasis from both primary and extrapulmonary tumors (236). Moreover, the disturbed and inefficient vascular supply of the TME and the elevated interstitial fluid pressure due to lymphovascular dysfunction greatly limit tumor penetration and T-cell infiltration into the tumor by dual antibodies. Small molecular weight dual antibodies (e.g., BiTE) that have better penetration but short half-lives (e.g., blinatumomab has a half-life of 2-4 hours (172)) require repeated administration. Small molecular weight dual antibodies (e.g., BiTE) that have better penetration but short half-lives (e.g., blinatumomab has a half-life of 2-4 hours (167)) require repeated administration. Fusing the VHH fragment of anti-HSA is a strategy that prolongs the drug’s half-life while maintaining high drug penetration, and several such bispecific antibodies are currently under clinical investigation. Finally, the current bsAbs are mainly targeted at some mature targets (e.g., EGFR, PD1, etc.), and breakthroughs are urgently needed in the mining of new lung cancer-specific targets (e.g., c-MET, HER3) and TME regulation strategies (e.g., combining with anti-angiogenic drugs).

### 7.5 Pharmacoeconomic considerations

In addition to the technical challenges discussed above, pharmacoeconomic considerations are also a key obstacle to the widespread application of bsAbs in treating lung cancer. Compared with conventional antibody drugs, bispecific antibodies have higher production costs and greater clinical development risks, resulting in higher drug prices. Moreover, adverse reactions caused by the drugs require additional symptomatic medications, which further increase the treatment costs for patients. Therefore, balancing the cost of the drug and its corresponding therapeutic effect is a huge challenge. Some researchers have noted that although the treatment regimens of amivantamab in combination with chemotherapy or amivantamab in combination with lazertinib provide significant benefits to patients, when the economic costs are considered, they are higher than the cost-effectiveness thresholds given current US pricing (237, 238). Therefore, how to balance the cost and pricing to benefit more patients is an issue that needs to be addressed. In addition, the treatment cost can be reduced by further optimising the drug dosage. For example, compared with the recommended treatment regimen, using an optimised alternative dosing regimen can save 16% of the treatment cost of Amivantamab (239).

## 8 Conclusion

BsAbs have ushered in significant development, with more than 110 in clinical development and nearly 180 in preclinical development (240). There are three drugs approved for lung cancer treatment, of which the launch of AMG757 is a breakthrough in the field of small-cell lung cancer treatment for many years, and AK112 beat Keytruda in head-to-head clinical studies, both of which are of landmark significance. In the next phase of drug development, the therapeutic potential of bsAbs will be further unlocked through research to optimize the production of CMC, reduce the adverse effects, and research into the pathogenesis of lung cancer itself, enhancing the cost-effectiveness of the drugs, which is of great significance, and discovering new therapeutic targets. In conclusion, more new drugs will be developed for more lung cancer patients with unmet needs for quite some time to come.
