# Correction: Bispecific antibody for lung cancer: mechanisms and clinical insights

**DOI:** 10.3389/fimmu.2025.1656082

**Published:** 2025-07-17

**Authors:** Wei Chen, Afang Zhou, Yunfeng Zhou

**Affiliations:** ^1^ Department of Thoracic Surgery, West China School of Public Health and West China Fourth Hospital, Sichuan University, Chengdu, Sichuan, China; ^2^ State Key Laboratory of Biotherapy, Sichuan University, Chengdu, Sichuan, China

**Keywords:** bispecific antibodies, lung cancer, novel therapies, immunotherapy, targeted therapy

In the published article, there was an error. The first sentence of the **Introduction** section contained erroneous data for the annual new lung cancer cases. The original text incorrectly stated “22 million new cases,” which should be “2.2 million new cases” as cited in Reference 1.

A correction has been made to the section **Introduction**, Paragraph 1. This sentence previously stated:

“Lung cancer is one of the world’s most common cancers and the leading cause of cancer-related deaths, with an estimated 22 million new cases and 1.79 million deaths annually (1).”

The corrected sentence appears below:

“Lung cancer is one of the world’s most common cancers and the leading cause of cancer-related deaths, with an estimated 2.2 million new cases and 1.79 million deaths annually (1).”

The original version of this article has been updated.

